# Comparative DNA Methylation Profiling Reveals an Immunoepigenetic Signature of HIV-related Cognitive Impairment

**DOI:** 10.1038/srep33310

**Published:** 2016-09-15

**Authors:** Michael J. Corley, Christian Dye, Michelle L. D’Antoni, Mary Margaret Byron, Kaahukane Leite-Ah Yo, Annette Lum-Jones, Beau Nakamoto, Victor Valcour, Ivo SahBandar, Cecilia M. Shikuma, Lishomwa C. Ndhlovu, Alika K. Maunakea

**Affiliations:** 1 Department of Native Hawaiian Health, John A. Burns School of Medicine, Suite 1016B, University of Hawaii, Honolulu, HI 96813, USA; 2 Department of Tropical Medicine, John A. Burns School of Medicine, University of Hawaii, 651 Ilalo Street, BSB325C, Honolulu, HI 96813, USA; 3 Hawaii Center for AIDS, John A. Burns School of Medicine, University of Hawaii, 651 Ilalo Street, BSB, Honolulu, HI 96815, USA; 4 Memory and Aging Center, Department of Neurology, University of California, San Francisco, CA, USA

## Abstract

Monocytes/macrophages contribute to the neuropathogenesis of HIV-related cognitive impairment (CI); however, considerable gaps in our understanding of the precise mechanisms driving this relationship remain. Furthermore, whether a distinct biological profile associated with HIV-related CI resides in immune cell populations remains unknown. Here, we profiled DNA methylomes and transcriptomes of monocytes derived from HIV-infected individuals with and without CI using genome-wide DNA methylation and gene expression profiling. We identified 1,032 CI-associated differentially methylated loci in monocytes. These loci related to gene networks linked to the central nervous system (CNS) and interactions with HIV. Most (70.6%) of these loci exhibited higher DNA methylation states in the CI group and were preferentially distributed over gene bodies and intergenic regions of the genome. CI-associated DNA methylation states at 12 CpG sites associated with neuropsychological testing performance scores. CI-associated DNA methylation also associated with gene expression differences including CNS genes *CSRNP1 (P* = 0.017), *DISC1 (P* = 0.012), and *NR4A2 (P* = 0.005); and a gene known to relate to HIV viremia, *THBS1 (P* = 0.003). This discovery cohort data unveils cell type-specific DNA methylation patterns related to HIV-associated CI and provide an immunoepigenetic DNA methylation “signature” potentially useful for corroborating clinical assessments, informing pathogenic mechanisms, and revealing new therapeutic targets against CI.

HIV-related cognitive impairment (CI) is characterized by debilitating impairments in cognitive, behavioral, and motor function with severity ranging from mild to severe (e.g. dementia)^1^. Diagnosis is based on abnormal performance on neuropsychological testing and can be further characterized in research settings by the presence or absence of functional limitations in daily activities. The prevalence of dementia due to HIV infection has drastically reduced since the advent of effective combination antiretroviral therapy (cART); however, less severe forms of CI occur in over 40% of chronically HIV-infected individuals despite long term cART^2^. Effective diagnostics and therapeutic options for HIV-related CI are limited. Research has focused on identifying biomarkers and uncovering biological mechanisms underlying the neuropathogenesis of HIV-related CI.

Many studies have focused on peripheral immune cells of the monocyte/macrophage lineage since these cells can be infected by HIV, functionally altered, and enter the central nervous system (CNS) either across a permeable blood brain barrier or potentially through the recently described meningeal lymphatic conduit^3^,^4^,^5^. Evidence suggests that monocytes traffic virus into the central nervous system^6^,^7^,^8^,^9^, infect resident microglial and astrocytes of the brain^10^, and induce a neuroinflammatory state^11^. This neuroinflammatory milieu is thought to activate neuronal apoptosis^12^,^13^,^14^ and underlie the development of impaired cognitive functioning^15^,^16^. Moreover, human studies suggest changes to cells of the monocyte/macrophage lineage influence the development of CI in the setting of HIV, as evidenced by the established associations between impaired cognitive performance and surface markers of CD14(+)CD16(+) monocytes^17^, monocyte activation markers in plasma^18^, and amount of HIV DNA in CD14(+) monocytes^19^. However, these studies have been inconsistent in identifying biomarkers linked to HIV-related CI, prompting the notion that epigenetic mechanisms may serve as a more reliable biomarker^20^. DNA methylation and histone modifications are two epigenetic mechanisms that determine and maintain cell identity and function^21^. Epigenetic mechanisms are dynamically responsive to environmental cues and research shows that HIV infection alters epigenetic processes in the immune system with changes to DNA methylation profiles of immune cells^22^,^23^. Whether a distinct DNA methylation profile associated with HIV-related CI exists in immune cell populations such as monocytes remains unknown.

Herein, we utilized genome-wide DNA methylation profiling to evaluate and compare the DNA methylation states of circulating monocytes in a cohort of HIV-infected participants based on clinical diagnostic criteria for the presence or absence of CI^24^. To determine whether the DNA methylation differences related to altered gene expression in monocytes, we performed genome-wide expression profiling of monocytes from impaired compared to unimpaired participants using targeted transcriptome sequencing. We subsequently performed genome-wide DNA methylation profiling of CD8+ T cells from individuals with CI compared to those without CI to investigate whether DNA methylation identified in monocytes could be observed in other immune cell types. We report novel evidence for epigenetic changes related to HIV-related CI in peripheral monocytes and CD8+ T cells.

## Results

### Classification of Cognitive Impairment

We examined cryopreserved peripheral blood mononuclear cells (PBMC) specimens from 21 HIV-infected individuals enrolled in the Hawaii Aging with HIV Cohort study classified as having CI based on meeting criteria for HIV-associated Dementia (HAD, n = 6) or Mild Cognitive Motor Disorder (MCMD, n = 5) using the American Academy of Neurology (AAN) 1991 as previously described (Supplemental Table S1)^24^. We randomly matched these CI cases with 10 HIV-infected control individuals with normal cognition (NL) based on education, gender, and age from the same cohort. There were no significant differences in main demographic and HIV clinical parameters between the two groups (Table 1). Neuropsychological testing performance differed by group using a composite measure of 8 tests (NPZ-8) with mean (SD) scores of 0.53 (0.43) and −1.18 (0.64) for impaired vs. unimpaired, respectively (*P* < 0.0001).

### Validation of monocyte cell enrichment by flow cytometry and cell type-specific DNA methylation analyses

Previous research has shown cellular heterogeneity of clinical blood samples can confound DNA methylation quantification and interpretations^25^. Hence, our DNA methylation analyses on bead-enriched monocyte populations were validated for monocyte enrichment by flow cytometry (Supplemental Figure S1). The flow cytometry based analysis of pre- and post-enrichment sample aliquots confirmed a significant post- compared to pre-enrichment for monocytes in both impaired (Mean Pre-: 15.8%; Mean Post-Enrichment: 90.3%, *P* < 0.0001) and matched non-impaired participants (Mean Pre-: 20.7%; Mean Post-Enrichment: 90.7%, *P* < 0.0001) (Supplemental Table S2). As an additional assessment of the cell purity, we accessed previously published DNA methylation of cell type-specific methylation markers from FACS-sorted human blood cells including CD14+ monocytes, CD8+ T cells, and PBMC^26^. We used this reference cell type-specific methylation dataset to examine the relationship between the reference methylation-specific CpG states for CD14+ monocytes and participants’ enriched monocyte samples at the same CpG sites. The monocyte cell type-specific methylation profile correlated with impaired (r = 0.89–0.98, *P* < 0.0001) and non-impaired (r = 0.87–0.97, *P* < 0.0001) monocyte samples post-enrichment methylation profile at the same CpG sites, providing evidence to confirm the enrichment of a majority cell population of monocytes (Supplemental Table S2). We also observed a correlation (r = 0.94, *P* <  0.0001) between percent monocyte enrichment values based on our FACS sorting results for the samples and the cell type-specific DNA methylation correlation (Supplemental Figure S2), suggesting this monocyte cell type-specific methylation profile could be used as a proxy of cell type composition analyses. These data support previous reports showing cell type-specific DNA methylation data can be used to corroborate estimates of cell populations^27^ and agrees with current attempts to adjust DNA methylation data for heterogeneous cell populations by estimating cellular proportions based on cell type-specific DNA methylation profiles^28^,^29^.

### Monocyte methylation signature and cognitive impairment

**Figure 1:**
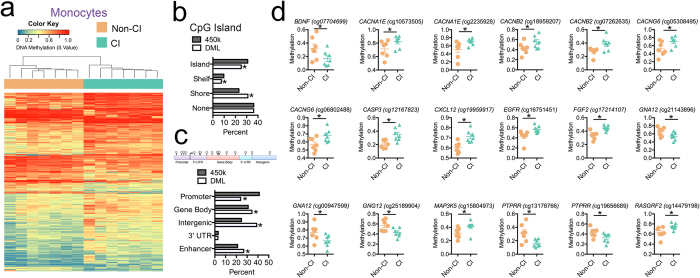
CI-associated DNA methylation differences in monocytes. (**a**) Heatmap displaying methylation levels of differentially methylated loci in Non-CI (orange) and CI (green) monocyte samples. Unsupervised hierarchical clustering analysis (Manhattan distance, complete linkage method) above columns identified 2 main clusters: Non-CI (orange) and CI (green). Methylation values displayed as ranging from low methylation (0, blue) to high methylation (1, red). (**b**) Plot showing percent differentially methylated loci (white) located in CpG islands, shelves, shores, or none compared to percent observed for all probes on the 450k array (grey). * Indicates significant difference. DML, differentially methylated loci. (**c**) Percent differentially methylated loci (white) located in gene promoters, gene bodies, intergenic regions, 3′ UTR, and enhancer regions compared to percent observed for all probes on the 450k array (grey) shows a significant enrichment for gene body, intergenic, and enhancer regions of the genome. UTR, untranslated region (**d**) Methylation of DML in Non-CI (orange) and CI (green) monocyte samples located in genes related to MAPK signaling. *P < 0.05.

To examine the genome-wide DNA methylation profile of monocytes between CI-diagnosed and non-CI groups, we acquired data of DNA methylation levels at single-nucleotide resolution using the Illumina Infinium HumanMethylation450 BeadChip (450 K) array. For the differential methylation analysis, we filtered probes showing absolute mean differences in methylation greater than 10% between CI-diagnosed and non-CI groups (Δβ-value > |0.1| at *P* < 0.05). This comparative analysis identified 1,032 differentially methylated CpG loci (DML) associated with CI (Dataset 1), with 71% of loci exhibiting hypermethylated states between CI and Non-CI groups. In agreement with recent reports, this hypermethylation phenotype of monocytes extends methylation findings observed in PBMCs comparing HIV-infected versus non-infected participants^30^. Next, we examined whether the differential methylation pattern in monocytes could distinguish between CI and Non-CI samples using unsupervised hierarchical clustering analysis of all samples. This analysis revealed a dendogram consisting of two clades stratifying all CI samples from Non-CI (Fig. 1a). In addition, we applied recursively partitioned mixture modeling (RPMM)^31^ of the monocyte DML and identified four methylation classes (Supplemental Figure S3). Among the four methylation classes, 2 classes perfectly captured only CI and 2 classes captured all Non-CI participants where class membership was significantly associated with CI (*P* = 0.0006) supporting the unsupervised hierarchical clustering analysis that stratified all CI samples from Non-CI (Fig. 1a).

Next, we evaluated whether the CI-associated DNA methylation differences preferentially occurred in specific genomic contexts. Specifically, our analyses examined whether the methylation differences were over CpG islands, gene promoters, gene bodies, or intergenic regions of the genome. We observed an enrichment of differentially methylated CpG sites located in CpG island shores, which are defined as sequences up to 2 kb from a CpG island (23.1% expected, 31.1% observed, *P* < 0.01; Fig. 1b). In comparison, we observed a lower than expected frequency of CpG sites located in CpG islands (30.9% expected, 25.9% observed, *P* < 0.01) and CpG shelf regions (9.7% expected, 7.5% observed, *P* < 0.01). The predominant differences were enriched in CpG sites located within gene bodies (30.92% expected, 34.4% observed, *P* < 0.01) and intergenic regions (24.7% expected, 38.8% observed, *P* < 0.01), but less than expected were located within gene promoters (41.2% expected, 23.9% observed, *P* < 0.01) (Fig. 1c). Since regulatory elements such as gene enhancers preferentially occur in gene body and intergenic regions, we examined whether the methylation differences we observed were enriched at annotated enhancer regions. As anticipated, differentially methylated CpGs were enriched in enhancer regions of the genome on the array as a whole (21.1% expected, 26.8% observed, *P* < 0.01; Fig. 1c), suggesting CI-associated differences of DNA methylation in monocytes may relate to epigenetic dysregulation of gene expression.

### CI-associated monocyte methylation signature related to the CNS and HIV infection

To examine which genes contained aberrant DNA methylation levels in CI compared to Non-CI samples, we first annotated the DML and identified 497 genes associated with these CpGs (Dataset 2). We used the Enrichr integrative web-based analysis tool to perform a gene list enrichment (amp.pharm.mssm.edu/Enrichr) of the 497 annotated genes associated with a DML^32^. The KEGG database analysis revealed enriched pathways related to the function of the central nervous system (CNS): neuroactive ligand receptor interaction (16 genes, *P* < 0.01, Table 2) and mitogen-activated protein kinase (MAPK) signaling (13 genes, *P* < 0.05, Fig. 1d); a molecular pathway implicated in regulation of HIV infectivity and latency^33^,^34^. Within the MAPK signaling pathway, we observed significant differences in methylation located at the stromal cell-derived factor-1 (*CXCL12*) gene (cg19959917: Non-CI = 0.605, CI = 0.715, *P* = 0.002), which interestingly is a ligand for HIV co-receptor CXCR4 and inhibits HIV-1 transmission by competing for CXCR4 binding^35^ (Fig. 1d). Moreover, CXCL12 is critical in chemotaxis and influences the migration of monocytes across the blood brain barrier^36^. Additionally, loci at the brain-derived neurotropic factor (*BDNF*) gene (cg07704699: Non-CI = 0.320, CI = 0.169, *P* = 0.03) and fibroblast growth factor 2 (*FGF2*) gene (cg17214107: Non-CI = 0.338, CI = 0.446, *P* = 0.001) were identified as differentially methylated in CI (Fig. 1d). Studies have reported altered expression of brain-derived neurotropic factor and fibroblast growth factors 1 and 2 in cerebrospinal fluid associate with HIV infection status and neurocognitive impairment^37^,^38^. In the neuroactive ligand receptor interaction pathway, we observed that *DRD3* harbored a CpG with significantly altered methylation levels in CI samples (cg25836326: Non-CI = 0.255, CI = 0.397, *P* = 0.015, Table 2). Previous studies have shown that a single nucleotide polymorphism within the dopamine related gene (*DRD3)* gene associates with neurocognitive impairments in HIV infected individuals dependent on methamphetamine^39^. The gene ontology (GO) enrichment results for biological process, cellular component, and molecular function confirmed CNS-related GO terms (Supplemental Table S3–S5). In addition to the gene list enrichment analysis of the differences in DNA methylation, we analyzed the genomic positions of all differentially methylated CpG sites using Genomic Regions Enrichment of Annotations Tool (GREAT) analysis^40^. GREAT analyzes the functional significance of *cis-*regulatory regions in the genome and assigns biological meaning to genomic locations. This analysis identified the top GO biological processes involved antigen processing and presentation of exogenous peptide antigen via MHC class I, regulation of T cell mediated cytotoxicity, and regulation of granule cell precursor proliferation (Supplemental Figure S4). Additionally, we identified 15 differentially methylated loci associated with CI in genes related to the inflammatory response (Supplemental Table S6), a biological process hypothesized in the pathogenesis of HIV-associated CI^41^. Taken together, these results suggest a CI-associated DNA methylation signature at genes involved in the CNS and HIV-related responses from the immune system including the inflammatory response.

Previous research has shown CI is associated with a higher viral load^42^ and higher circulating cell-associated HIV DNA^43^ suggesting a difference in the host immune system’s response to the virus in CI. To determine if the CI-associated differences in DNA methylation related to proteins known to interact with HIV, we analyzed the CI-associated differences in DNA methylation against curated genomic data repositories using VirusMINT^44^. The VirusMINT database contains all protein interactions between viral and human proteins. This analysis revealed 12 genes containing a DML that were associated with a curated HIV interaction (Table 3), suggesting changes in DNA methylation levels might represent a molecular mechanism by which monocytes respond differentially to HIV infection and might be relevant to CI pathogenesis. For example, the expression of the *HLA-G* gene was previously shown to be higher in monocytes from HIV participants^45^ and certain genetic variants associate with protection from HIV infection^46^. We identified that a CpG located in the transcriptional start site of the *HLA-G* gene was 32% significantly lower in methylation in CI compared to Non-CI samples (Table 3). These data suggest monocytes from those individuals with CI harbor distinguishing epigenetic profiles related to antigen presentation and HIV infection, which may confer an increased vulnerability to HIV-associated disorders such as CI.

### Relationship between CI-associated DNA methylation and neuropsychological function

**Figure 2:**
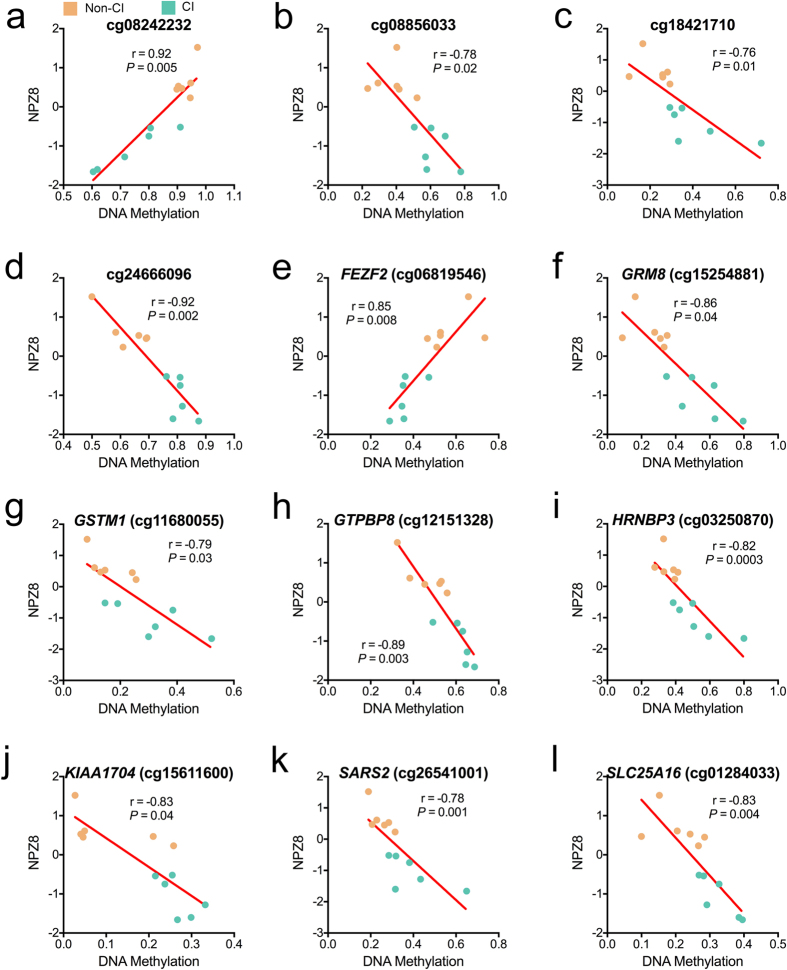
Relationship of CI-associated DNA methylation and neuropsychological test score. (**a–l**) Correlations between DNA methylation levels at specific genomic loci and neuropsychological testing data. NPZ8, Transformed z-scores measuring global neuropsychological performance. Non-CI data displayed in orange and CI in green. Benjamini-Hochberg adjusted *P*-value.

To examine whether DNA methylation of monocytes associated with neuropsychological function, we determined the correlations between individual’s NPZ8 global neuropsychological score and the CI-associated DML identified in monocytes. After determining the correlation between NPZ8 and DNA methylation levels at all DML and correcting for multiple correlations with the Benjamini-Hochberg procedure, we identified 12 significant (*P* < 0.05) relationships (Fig. 2). Four of the significant relationships occurred at CpGs without an associated gene transcript (cg08242232: r = 0.92, *P* = 0.005; cg08856033: r = −0.78, *P* = 0.02; cg18421710: r = −0.76, *P* = 0.01; and cg24666096: r = −0.92, *P* = 0.002). A positive relationship was identified between higher levels of methylation and increased neuropsychological function at a CpG (cg06819546: r = 0.85, *P* = 0.008) in the CNS linked gene FEZ Family Zinc Finger 2 (*FEZF2)*. In contrast, we identified a negative relationship between higher levels of methylation and decreased neuropsychological function at CpGs (Fig. 2) for Glutamate Receptor, Metabotropic 8(*GRM8)* (cg15254881: r = −0.86, *P* = 0.04), Glutathione S-Transferase Mu 1(*GSTM1)* (cg11680055: r = −0.79, *P* = 0.03), GTP-Binding Protein 8(*GTPBP8)* (cg12151328: r = −0.89, *P* = 0.003), RNA Binding Protein, Fox-1 Homolog(*HRNBP3)* (cg03250870: r = −0.82, *P* = 0.0003), Lipopolysaccharide Specific Response-7 Protein(*KIAA1704)* (cg15611600: r = −0.83, *P* = 0.04), Seryl-TRNA-Synthetase 2, Mitochondrial(*SARS2)* (cg26541001: r = −0.78, *P* = 0.001)*, and* Solute Carrier Family 25 Mitochondrial Carrier Member 16(*SLC25A16)* (cg01284033: r = −0.83, *P* = 0.004). These results suggest specific DNA methylation signatures of monocytes may have potential utility as a prospective biomarker or provide information for assessing the severity of CI in HIV.

### CI-associated monocyte methylation signature relates to altered gene expression

**Figure 3:**
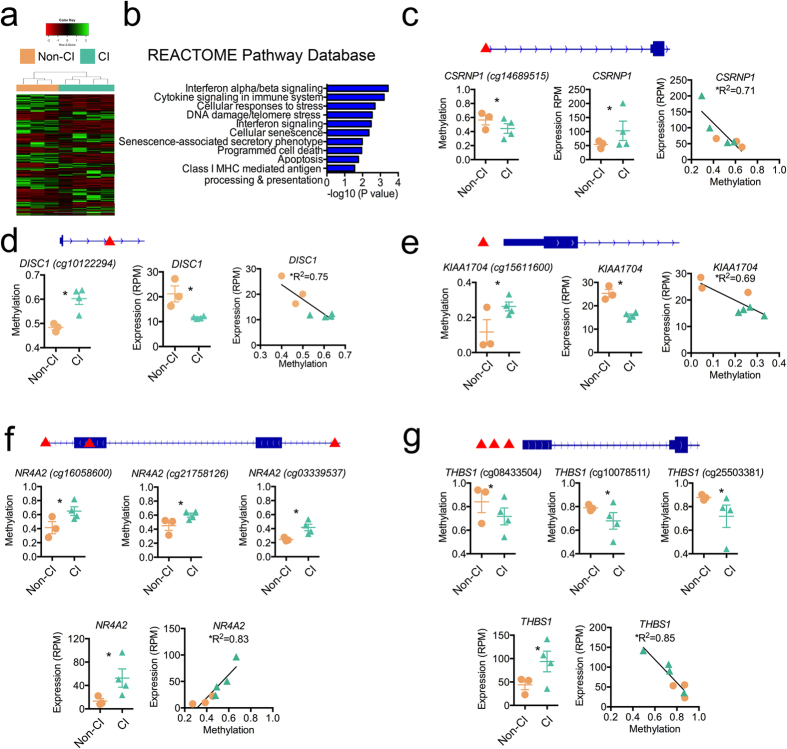
Relationship of CI-associated DNA methylation and gene expression in monocytes. (**a**) Heatmap of 496 differently expressed genes comparing Non-CI (orange) and CI (green) monocyte samples. Unsupervised hierarchical clustering analysis (Manhattan distance, complete linkage method) above columns identified 2 main clusters: Non-CI (orange) and CI (green). Expression values displayed ranging from low expression (red) to high expression (green). (**b**) Gene enrichment of differentially expressed genes from REACTOME Pathway Database. (**c**) CI-associated DNA methylation significantly associated with altered expression of *CSRNP1* gene (r = −0.84, *P* = 0.01), (**d**) *DISC1* gene (r = −0.87, *P* = 0.01), (**e**) *KIAA1704* gene (r = −0.83, *P* = 0.02), (**f**) *NR4A2* gene (r = 0.91, *P* = 0.004), and (**g**) *THBS1* gene (r = −0.92, *P* = 0.003). (**c–g**) Gene structures displayed in blue above graphs and location of CpG sites indicated by red arrows. Methylation values presented as β-values and expression quantified as reads per million (RPM). Orange circle (Non-CI) and green triangle symbols (CI).

DNA methylation in specific regions of the human genome (e.g., gene promoters) has been shown to associate with transcription^47^ and previous studies have reported altered gene expression in HIV-associated neurocognitive disorders^48^. Therefore, to examine whether the observed CI-associated DNA methylation differences at 497 genes related to expression differences, we examined genome-wide expression in a subset of monocyte samples from CI and Non-CI participants for which we examined DNA methylation. We performed targeted transcriptome sequencing using the Ion Torrent semiconductor sequencing platform to measure gene expression levels of 20,802 RefSeq genes. We obtained an average of 8.6 million reads per sample with 92.7% of reads aligned over target coding gene regions and passing filters for minimum alignment length. From this dataset, we identified 496 genes that exhibited significant differences in expression levels between CI and Non-CI samples (Fig. 3a). Interestingly, pathway analysis of these differentially expressed genes revealed the top pathways (Fig. 3b) were related to interferon signaling and cytokine signaling (Supplemental Table S7) in the immune system. These findings support previous reports of altered gene expression of genes involved in the interferon signaling pathway in postmortem brain tissue of HAND samples^48^ and genes involved in inflammation in HIV-infected peripheral blood monocytes that associated with neurocognitive functioning^49^. Moreover, these results support our findings of CI-associated differences in DNA methylation states of genes involved in the inflammatory response (Supplemental Table S6), which are related to interferon signaling and cytokine signaling pathways.

Next, we integrated the expression and methylation datasets for which we had matched samples (n = 7) and examined the overlap of differentially expressed (496) and methylated (497) genes. This analysis identified only 5 genes (1%) were differentially expressed and contained a differentially methylated CpG loci. The Cysteine-Serine-Rich Nuclear Protein 1(*CSRNP1*) gene contained a differentially methylated CpG in a region proximal to but downstream of the promoter (Fig. 3c). The significant decrease in methylation in CI compared to Non-CI for this gene was inversely correlated with a significant increase in gene expression (r = −0.84, *P* = 0.017). *CSRNP1* encodes a protein involved in the Wnt signaling pathway^50^ and binds to a consensus sequence with transcriptional activator activity with a role in apoptosis. Another Wnt signaling pathway related gene, Disrupted in Schizophrenia 1 (*DISC1*) contained a hyper-methylated CpG (Fig. 3d) significantly associated with decreased gene expression in CI compared to Non-CI (r = −0.87, *P* = 0.012). *DISC1* encodes a protein that is critical to brain function and genetic variants of this gene have been associated with neurocognitive deficits in schizophrenia^51^. Moreover, expression of DISC1 in peripheral blood has been shown to be a correlate of cognitive performance and prefrontal cortex brain activity in schizophrenia^52^. The *KIAA1704* gene contained a differentially methylated CpG in the promoter region of the gene (Fig. 3e) significantly associated with decreased gene expression in CI compared to Non-CI (r = −0.83, *P* = 0.021). Moreover, for another overlapping gene, Nuclear Receptor Subfamily 4, Group A, Member 2(*NR4A2),* we observed a cluster of 3 significantly hypermethylated CpGs in the gene body region in CI compared to Non-CI, suggestive of a differentially methylated region (Fig. 3f). These CpGs were positively associated with increased gene expression in CI compared to Non-CI (r = 0.91, *P* = 0.005). This positive association between methylation and gene expression supports previous reports of DNA methylation’s role in gene bodies related to the regulation of alternative splicing^47^,^53^. *NR4A2* regulates dopamine neurons in the brain during development^54^, differentiation of CD4 T cells^55^, and mutations in this gene have been associated with degenerative brain related disorders including Parkinson’s disease, schizophrenia, and depression^56^,^57^. For the Thrombospondin 1(*THBS1)* gene, we identified 3 CpG loci significantly hypomethylated in CI compared to Non-CI individuals (Fig. 3g) located near the transcription start site (r = −0.92, *P* = 0.003). Confirming the widely accepted relationship between gene promoter methylation and expression, we observed significantly higher DNA methylation at the gene promoter of *THBS1* in CI compared to Non-CI was related to significantly lower expression (Fig. 3g). *THBS1* encodes a glycoprotein that mediates cell-to-cell and cell-to-matrix interactions. Interestingly, levels of *THBS1* have been shown to associate with HIV elite suppressors^58^. Our integrated analyses of CI-associated DNA methylation and gene expression differences reveal a subset of differentially expressed genes in monocytes that are potentially under epigenetic regulation.

### Cross cell-type replication of CI-associated CpGs in CD8+ T Cells

We determined whether the CI-associated differential DNA methylation profile and perturbed biological processes identified in monocytes are distinct to this cell type or manifest in other immune cell types. Given CD8+ T cells have been associated with and proposed to play a role in the pathogenesis of CI^59^, we enriched for CD8+ T cells from previously frozen PBMCs of a subset of individuals (n = 7) with CI and with no cognitive impairment (Non-CI) and investigated whether a distinct methylation profile differentiated CI CD8+ T cells from Non-CI participants. We confirmed our enrichment for CD8+ T cells by a cell type-specific DNA methylation analysis (Supplemental Table S8) and identified 1,431 differentially methylated loci (Δβ-value > |0.1| at *P* < 0.05) comparing CI versus Non-CI samples.

**Figure 4:**
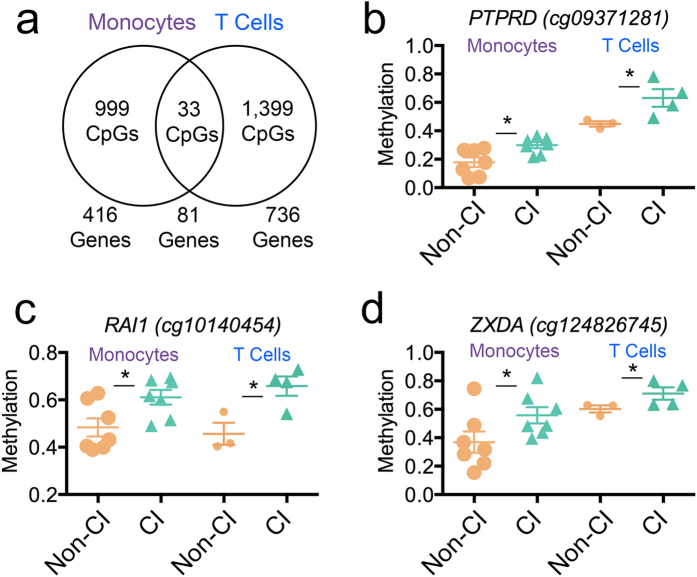
Cross cell-type replication of CI-associated CpGs in CD8+ T Cells. (**a**) Venn diagram showing frequency of CI-associated differentially methylated loci and genes observed in monocytes (purple) that overlap in T cells (blue). (**b**) DNA methylation is significantly higher in CI (green) monocytes and T cells compared to Non-CI (orange) for a CpG in the *PTPRD*, (**c**) *RAI1*, and (**d**) *ZXDA* genes. * denotes *P*-values < 0.05.

**Figure 5:**
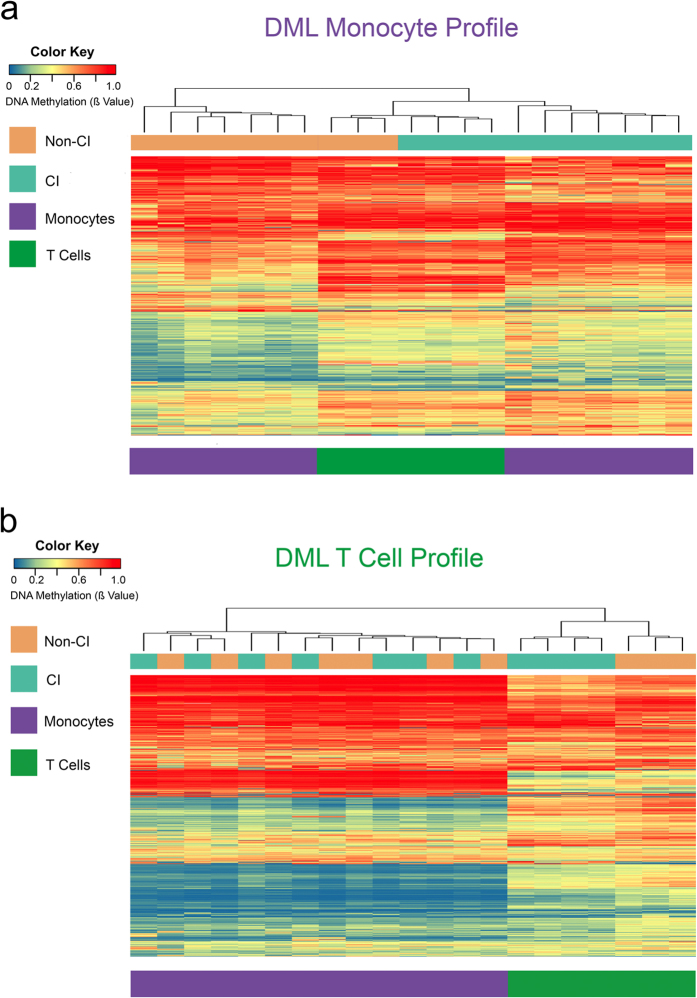
CI-associated DML in monocytes stratifies CI in T cells. Heatmap displaying methylation levels of differentially methylated loci in Non-CI (orange) and CI (green) monocyte (purple bar) and T cell (green bar) samples. (**a**) Using monocyte DMLs, unsupervised hierarchical clustering (Manhattan distance, complete linkage method) above columns shows monocytes stratify from T cells in 3 distinct clades and Non-CI stratifies from CI samples. In contrast, (**b**) using T cell DMLs, unsupervised hierarchical clustering above columns only shows monocytes stratify from T cells, but Non-CI does not stratify from CI samples in monocytes. Methylation values displayed as ranging from low methylation (0; blue) to high methylation (1, red).

Next, we examined whether the methylation differences overlapped in CD8+ T cells by integrating the CD8+ T cell DML with the monocyte DML. This analysis revealed 81 genes (16.3%) and 33 CpGs (3.2%) overlapped (Fig. 4a), indicating a proportion of the CI-associated DNA methylation signature detected in monocytes was detectable in CD8+ T cells. For example, CpG loci at the genes Protein Tyrosine Phosphatase, Receptor Type, D (*PTPRD)*, Retinoic Acid Induced 1(*RAI1)*, and Zinc Finger, X-Linked, Duplicated A (*ZXDA)* were significantly hypermethylated in CI compared to Non-CI from both monocytes and CD8+ T cells (Fig. 4b–d). *PTPRD* has been implicated in regulating neuron axon guidance and *ZXDA* has been shown to promote transcription of MHC class I and II genes. The *RAI1* gene is highly expressed in neuronal tissues and may function as a transcriptional regulator through chromatin remodeling. We next examined using unsupervised hierarchical clustering whether the 1,032 DML identified from the monocyte profile in Fig. 1a could stratify CI participants from non-CI participants independent of cell type using the DML in monocytes to cluster the CD8+ T cells. Surprisingly, this clustering analysis identified all CI stratified from all non-impaired participants in both monocytes and T cells (Fig. 5a). Next, we used the CD8+ T cell DML (1,431 CpGs) to see if a similar stratification could occur independent of cell type. In contrast to the monocyte DML signature, this clustering and recursively partitioned mixed modeling analyses revealed the CD8+ T cell profile did not stratify CI and Non-CI monocyte samples (Fig. 5b, Supplemental Figure S5). This result suggests monocytes may harbor a non-cell type specific CI-associated immunoepigenetic signature, which may serve as a biomarker. Future work will need to examine this hypothesis and the interactions of monocytes and T cells in the context of HIV-related CI. Moreover, these findings indicate a subset of DNA methylation states at specific loci that are CI-associated are independent of cell type and may be detectable in heterogeneous populations of peripheral immune cells such as PBMCs, and may be potentially useful as a immunoepigenetic biomarker for CI.

Since the majority of CI-associated monocyte loci did not overlap with CD8+ T cells, we examined these genes and biological processes identified in CD8+ T cells. This analysis revealed CI participants could be stratified from Non-CI based on differences in CD8+ T cell methylation states (Supplemental Figure S6a). Additionally, supporting the monocyte methylation results, we observed a significant enrichment of DML in CpG island shores and gene body and intergenic regions (Supplemental Figure S6b,c). Interestingly, the top GO biological processes included interferon gamma mediated signaling, and regulation of adaptive immune response (Supplemental Figure S6d). In particular, we observed DML at genes involved in HIV infection including *CCR5, CXCR4*, and *IFNG* in CD8+ T cells from the 1,431 differentially methylated loci comparing CI versus Non-CI samples (Supplemental Figure S7). This is intriguing given that mouse studies revealed genetic ablation of CCR5 prevented microglial and neuronal damage of HIV-associated brain injury induced by a CXCR4-using viral envelope gp120^60^. Moreover, we identified DML at genes implicated in neurocognitive disorders such as *APBB1*, *BRCA1, DNMBP* and *MAPT, NCAM1*; and epigenetic mechanism-related genes including *BCOR, DNMT3A, and HDAC4* (Supplemental Figure S7). These novel findings extend the CI-associated monocyte methylation results and suggest a unique immunoepigenetic signature of CI in CD8+ T cells. Additionally, these findings provide strong support for further studies examining epigenetic mechanisms in CD8+ T cells and other immune cell subsets involved in HIV infection and disease progression related to CI.

## Discussion

Monocytes are widely accepted as playing a crucial role in the pathogenesis of HIV-related CI. Here we provide evidence for a distinct differential methylomic signature in peripheral monocytes obtained from well-characterized HIV infected individuals stratified on the basis of CI. We identified differentially methylated CpG sites with a greater than 10% difference at specific loci between CI and Non-CI individuals related to genes involved in the CNS and interacting with HIV. These CI-associated methylation differences occurred preferentially at regulatory regions of the genome including CpG island shores, gene bodies, intergenic, and enhancer regions. In addition, we observed CI-associated methylation differences associated with gene expression and neuropsychological test scores. Also, a fraction of the CI-associated methylation differences observed in monocyte cells were independently replicated in T cells. Together, these findings support previous studies highlighting monocyte perturbations associated with CI^17^ and suggest an immunoepigenetic signature of CI may exist.

HIV proteins affect the epigenotype and transcriptomes of host cells^61^,^62^. Specifically, DNA methyltransferease activity is altered in T cells following HIV infection^61^. These HIV-induced epigenetic changes occur at the early stages of HIV infection; however, little is known about the dynamic changes that occur throughout the course of infection and those that relate to HIV-related comorbidities including CI. Our findings address this gap in knowledge and provide correlative evidence of a specific immunoepigenetic signature of individuals with HIV-related cognitive impairment. However, a key question that needs to be pursued is what possible mechanisms may be driving the DNA methylation differences observed in specific cell types isolated from HIV patients diagnosed with or without CI. We suspect the DNA methylation differences observed in monocytes and T cells isolated from HIV patients diagnosed with or without CI may arise from differences in the HIV reservoir. Recent results show that HIV replication persist in tissue reservoirs during therapy in patients with undetectable levels of virus in the bloodstream^63^. Differences among individuals in HIV replication and replenishment of the viral reservoir may provide the underlying driver of changes in DNA methylation in immune cell types in HIV-related CI. Future work is required to test this possible hypothesis and understand the functional consequences of the differences in monocyte activity due to epigenetic alterations.

Interesting clues into the neuropathogenesis of HIV-related CI can be gleaned from the integrative analysis of differentially methylated loci related to altered gene expression observed in our study. Surprisingly, we observed that three of the five CI-related DNA methylation and associated expression differences detected in monocyte cells isolated from the periphery of HIV participants related to the central nervous system. A possible mechanism linking the CI-related epigenetic differences of DNA methylation and gene expression in peripheral immune cells to the neuropathogenesis of CI is active monocyte trafficking of this dysregulated epigenetic state at CNS-related genes into the brain. Studies suggest that epigenetic states of brain microglia may be modified by exogenous states^64^. This may occur directly through the recently described CNS lymphatic system^5^, which would allow immune cells to easily enter and exit the CNS. We also suspect that an epigenetic dysfunction in peripheral immune cells of certain individuals at risk for developing HIV-related CI may interact and confer a similar epigenetic state to brain resident cells such as microglia cells, which may drive the brain dysfunction. Future work will need to determine whether a dysregulated epigenetic state in peripheral immune cells can actively influence epigenetic states in the CNS and whether this is involved in the neuropathogenesis in patients with CI.

Several limitations exist for this study including the application of the Illumina methylation array (450k) technology to profile the methylome of these clinical samples at single nucleotide resolution^65^. This methodology has been used in previous studies examining HIV infected brain and tissue specimens^22^; however, this DNA methylation assay is limited to surveying approximately 1.7% of the total CpG sites within the human genome and biased to specific genomic regions. To identify other regions of the genome containing methylation differences, additional studies may employ techniques offering more extensive genome coverage of CpGs, such as whole genome bisulfite sequencing experiments or the MeDIP method^66^. Another limitation of the study is the small number of subjects. We limited our analysis to the monocyte cell type using immunomagnetic separation to eliminate variability due to cellular heterogeneity, and suspect this allowed us to detect robust changes in DNA methylation states associated with clinical features even with small sample size. These results should be interpreted with caution and need to be replicated in a larger sample size and across different immune cell subtypes to further support the existence of an immunoepigenetic signature of HIV-related CI. Future studies may therefore consider either isolating pure populations of subsets of monocytes to profile or examine whether single cell level analyses reveals differences among monocyte subsets previously characterized^29^,^67^. In summary, this study supports the hypothesis that an altered DNA methylation state of monocyte/macrophage contributes to CI, and suggests epigenetic profiles in specific immune cell types may serve as a biomarker of the disorder.

## Methods

### Sample Cohorts and Clinical data

Demographic and clinical data for participants were compared using nonparametric Wilcoxon-Mann-Whitney test. Informed consent was obtained from participants following procedures approved by the University of Hawaii Human Studies Institutional Review Board. All experiments were performed in accordance with relevant guidelines and regulations.

### PBMC Specimens, Monocyte/T cell Enrichment, and Nucleic Acid Isolation

Viably cryopreserved peripheral blood mononuclear cells (PBMCs) specimens (4–6 million cells) were thawed using AIM-V Serum Free Medium (Thermo Fisher) supplemented with 2% DNase, washed, and resuspended in buffer consisting of PBS, 3% BSA, and 1 mM EDTA. An aliquot of all PBMCs were stained and quantified using the Countess Automated Cell Counter (Life technologies). PBMCs were used to negatively select for monocytes (CD14+) by magnetic bead separation (EasySep Human Monocyte Enrichment Kit without CD16 depletion) or T cells (EasySep Human CD8+ T Cell Enrichment Kit) according to manufacturer’s instructions (StemCell Technologies). DNA and RNA were isolated from enriched monocytes or T cells using the AllPrep DNA/RNA kit (Qiagen) according to the manufacturer’s recommendations for cells. Nucleic acid concentrations were determined using the Qubit DNA Broad Range or RNA Broad Range fluorescence assays (Life Technologies) and Qubit Instrument (Life Technologies).

### FACS Validation of Monocyte Enrichment

To confirm the enrichment for monocytes by negative selection, an aliquot (125,000 cells) for all subjects pre- and post-enrichment was analyzed by a flow cytometer for monocytes, T cells, NK cells, and B cells (Supplemental Figure S1). Aliquots were stained with yellow amine fluorescent reactive dye (Life Technologies), anti-CD16 Brilliant Violet 421 (Clone 3G8), anti-CD3 V500 (Clone UCHT1), anti-CD14 Qdot605 (Clone TuK4), anti-CD56 Pe-Cy7 (Clone B159), anti-CD19 PE-Cy7 (Clone 1D3), anti-CD20 Pe-CY7 (Clone 2H7), and anti-HLA-DR APC-H7 (Clone G46-6). Anti-mouse IgG/Negative Control (FBS) Compensation Particle Set (BD Bioscience) was used for compensation of sample data for normalization. Anti-mouse IgG compensation beads were stained with each fluorochrome-conjugated antibodies in separate wells. Stained cells from PBMCs and isolated monocyte samples were analyzed using a 4-laser BD LSRFortessa flow cytometer (BD Bioscience). Compensation and gating analyses used FlowJo software (Tree Star, Inc.). The gating strategy for identification of monocytes was according to previous reports^68^. Briefly, monocytes were identified by excluding dead cells, lymphocytes (CD3+) natural killer cells (CD56+), and B Cells (CD19+ or CD20+). Monocytes were taken as HLA-DR+ and subsetting by CD14 and CD16 expression. The cell frequency (%) was determined by event count (specific event/total events). The average purity of monocytes post-enrichment was 90.3% for CI and 90.7% for Non-CI with minimal contamination by other cellular populations.

### Cell Type-Specific Differential Methylation Validation of Monocytes and CD8+ T Cell Enrichment

Illumina HumanMethylation450 BeadChip data was downloaded from GEO accession: GSE35069^26^. Cell type-specific methylation data for FACS-sorted CD14+ monocytes (n = 6), CD8+ T cells (n = 6), and PBMC samples (n = 6) were used to determine cell type-specific DNA methylation sites. We used this dataset to examine the relationship to monocyte- and T cell-enriched CI and Non-CI samples methylation.

### Illumina 450k Array-based DNA Methylation Analysis

500 ng of DNA per sample were bisulfite converted using the EZ DNA Methylation kit (Zymo Research) according to the manufacturer’s instructions. Bisulfite-converted DNA samples were randomly assigned to a chip well on the Infinium HumanMethylation450 BeadChip, amplified, hybridized onto the array, stained, washed, and imaged with the Illumina iScan SQ instrument to obtain raw image intensities at the University of Hawaii Cancer Center Genomics Shared Resource. Raw Methylation array IDAT intensity data was preprocessed in R statistical programming language (http://www.r-project.org) using the RnBeads 0.99.18 pipeline analysis package^69^ to filter probes (detection p-values > 0.05, missing probes, non-specific probes, and SNP-enriched probes) and samples that could bias the normalization procedure. Methylation β-values ranging from 0–1 (corresponding to unmethylated to methylated signal intensity) for each sample were normalized using the subset-quantile within-array normalization (SWAN) method^70^ option implemented in the minfi package and available in the RnBeads pipeline. Differential methylation analysis was conducted on the site level using linear models employed in the limma R package. Sites were identified as significant (p < 0.05) and filtered for sites with absolute methylation differences greater than 10% (Δβ-value) between groups. Differentially methylated probes were annotated using the 450 k array v1.2 annotation (Illumina). Region enrichment analysis of distribution of CpG sites for gene location and CpG island regions was performed using chi-square goodness of fit tests with each category vs. sum of all other categories with Bonferroni correction. The Recursively Partitioned Mixture Model (RPMM) R package v1.2 was used for methylation clustering and analysis of associations between methylation class (categorical) and individual categorical variable Non-CI or CI was performed using Fisher’s exact test.

### Targeted Human Transcriptome Profiling

The Ion AmpliSeq Transcriptome Human Gene Expression panel (Life Technologies) was utilized for gene expression profiling of monocytes. The panel uses targeted sequencing of 20,802 RefSeq genes. 100 ng of total RNA was used from enriched monocytes of CI and Non-CI to construct sequencing libraries according to manufacturer’s instructions. Indexed sequencing libraries were constructed using the Human Transcriptome AmpliSeq kit and quantified using the Ion Library Quantification kit on a StepOnePlus Real-Time PCR system. Eight multiplexed samples were templated using the Ion P1 Template OT2 200 Kit V3 with Ion OneTouch 2 Instrument, enriched, loaded on an Ion P1 semiconductor sequencing chip, and run on the Ion Proton Semiconductor Sequencer using the Ion P1 Sequencing 200 Kit V3. The Ion TorrentSuite software was used to map reads and the Ion ampliSeqRNA plugin was used to attain normalized read count data files (RPM) for each sample. Differential gene expression statistical analysis was performed on normalized (Reads Per Million reads) expression values assigned to each amplicon for all samples using the R statistical programming language package DESeq, which estimates variance-mean dependence in count data and tests for differential expression using a model based on negative binomial distribution.

## Additional Information

**How to cite this article**: Corley, M. J. *et al.* Comparative DNA Methylation Profiling Reveals an Immunoepigenetic Signature of HIV-related Cognitive Impairment. *Sci. Rep.* **6**, 33310; doi: 10.1038/srep33310 (2016).

## Supplementary Material

Supplementary Dataset 1

Supplementary Dataset 2

Supplementary Information

## Figures and Tables

**Table 1:** Participant Characteristics for Comparative DNA Methylation Profiling.

	Non-CI (n = 10)	CI (n = 11)	
Gender (% Male)	86%	86%	
Ethnicity (% Caucasian)	80%	82%	
Education (Years)	14 (12, 16)	15 (12, 18)	*P* = 0.05
Age (Years)	55 (50, 60)	58 (51, 72)	*P* = 0.36
CD4 Nadir	327 (34, 850)	385 (8, 900)	*P* = 0.61
CD4 Count	456 (160, 853)	573 (155, 1352)	*P* = 0.64
Neuropsychological Composite score (NPZ-8)	0.53 (0.01, 1.52)	−1.18 (−0.42, −2.46)	*P* < 0.0001
Viral Load (Log_10_)	2.9 (1.7, 5.0)	1.9 (1.7, 3.6)	*P* = 0.10
	Mean (Min, Max)	Mean (Min, Max)	

^*^P-values calculated by Wilcoxon-Mann-Whitney test.

**Table 2:** Monocyte DML: KEGG 2015 Neuroactive Ligand Receptor Interaction.

Probe ID	Genomic Position	Gene	Gene Region	*P*-value	Mean Non-CI	Mean CI	Beta Diff.
cg25836326	chr3:113898609-113898610	*DRD3*	TSS1500	0.02	0.26	0.40	0.14
cg11335335	chr11:637885-637886	*DRD4*	Body	0.01	0.25	0.40	0.15
cg07212818	chr11:638076-638077	*DRD4*	Body	0.03	0.49	0.63	0.13
cg01616529	chr11:638424-638425	*DRD4*	Body	0.04	0.53	0.69	0.16
cg17401282	chr5:161494274-161494275	*GABRG2*	TSS1500	0.02	0.41	0.27	0.13
cg11494091	chr17:61959527-61959528	*GH2*	TSS1500	0.04	0.83	0.71	0.12
cg00901598	chr3:172164984-172164985	*GHSR*	Body	0.02	0.71	0.59	0.12
cg04851268	chr3:172167445-172167446	*GHSR*	TSS1500	0.03	0.24	0.35	0.10
cg25352328	chr5:151304817-151304818	*GLRA1*	TSS1500	0.01	0.23	0.40	0.17
cg26738987	chr11:94135029-94135030	*GPR83*	TSS1500	0.01	0.38	0.50	0.12
cg14351882	chr9:140061878-140061879	*GRIN1*	Body	0.01	0.23	0.37	0.13
cg17867333	chr5:178423163-178423164	*GRM6*	TSS1500	0.00	0.70	0.81	0.11
cg15254881	chr7:126890512-126890513	*GRM8*	5′UTR	0.00	0.24	0.55	0.30
cg02918903	chrX:114141866-114141867	*HTR2C*	Body	0.02	0.40	0.59	0.19
cg27551227	chr7:154877217-154877218	*HTR5A*	3′UTR	0.01	0.71	0.52	0.18
cg20967585	chr7:154862524-154862525	*HTR5A*	TSS200	0.01	0.25	0.36	0.11
cg06823034	chr14:24780734-24780735	*LTB4R*	TSS200	0.01	0.09	0.19	0.11
cg12600858	chr11:92702530-92702531	*MTNR1B*	TSS1500	0.04	0.25	0.35	0.11
cg03873322	chr6:142409202-142409203	*NMBR*	Body	0.03	0.37	0.26	0.11
cg11881038	chr6:154408701-154408702	*OPRM1*	5′UTR	0.00	0.58	0.91	0.33

**Table 3:** Monocytes DML: VirusMINT Human Immunodeficiency Virus 1.

Probe ID	Genomic Position	Gene	Gene Region	*P*-value	Mean Non- CI	Mean CI	Beta Diff.
cg16999994	chr11:1001560-1001561	*AP2A2*	Body	0.02	0.66	0.40	−0.25
cg14351882	chr9:140061878-140061879	*GRIN1*	Body	0.01	0.23	0.37	0.13
cg01790498	chr7:44888626-44888627	*H2AFV*	TSS1500	0.02	0.34	0.45	0.11
cg18009000	chr10:71811927-71811928	*H2AFY2*	TSS1500	0.03	0.83	0.72	−0.11
cg25372449	chr6:32490350-32490351	*HLA-DRB5*	Body	0.04	0.47	0.70	0.23
cg25046571	chr6:29794657-29794658	*HLA-G*	TSS200	0.03	0.83	0.50	−0.32
cg24686153	chr15:56148763-56148764	*NEDD4*	Body	0.04	0.70	0.80	0.10
cg08914678	chr16:334889-334890	*PDIA2*	Body	0.02	0.81	0.63	−0.18
cg01309213	chr16:331821-331822	*PDIA2*	Body	0.03	0.74	0.63	−0.11
cg09388991	chr8:30669313-30669314	*PPP2CB*	Body	0.02	0.80	0.69	−0.11
cg10077239	chr14:30397686-30397687	*PRKD1*	TSS1500	0.00	0.30	0.41	0.11
cg08161802	chr1:31377381-31377382	*SDC3*	Body	0.05	0.36	0.46	0.10
cg25503381	chr15:39871923-39871924	*THBS1*	TSS1500	0.01	0.89	0.77	−0.12
cg08433504	chr15:39872071-39872072	*THBS1*	TSS1500	0.02	0.87	0.75	−0.12
cg10078511	chr15:39872032-39872033	*THBS1*	TSS1500	0.02	0.83	0.73	−0.10
