# Supplementary material for: Comparative DNA Methylation Profiling Reveals an Immunoepigenetic Signature of HIV-related Cognitive Impairment
Source: Sci Rep. 2016 Sep 15;6:33310. doi: 10.1038/srep33310 (PMC5024304; doi:10.1038/srep33310)
Supplement: Supplementary Information [file srep33310-s3.pdf]

Comparative DNA Methylation Profiling Reveals an Immunoepigenetic Signature of HIV-related Cognitive Impairment

Michael J. Corley[1], Christian Dye[1], Michelle D'Antoni Brogan[2], Mary Margaret Byron[2], Kaahukane Leite-Ah Yo[1], Annette Lum-Jones[1], Beau Nakamoto[3], Victor Valcour[4], Ivo Sah Bandar[2], Cecilia M. Shikuma[3], *Lishomwa C. Ndhlovu[2,3,], and *Alika K. Maunakea[1]

[1]Department of Native Hawaiian Health, John A. Burns School of Medicine, Suite 1016B, University of Hawaii, Honolulu, HI 96813, USA.

[2]Department of Tropical Medicine, [3]Hawaii Center for AIDS, John A. Burns School of Medicine, University of Hawaii, 651 Ilalo Street, BSB, Honolulu, HI 96815, USA,

[4]Memory and Aging Center, Department of Neurology, University of California, San Francisco, CA, USA

Supplementary Information

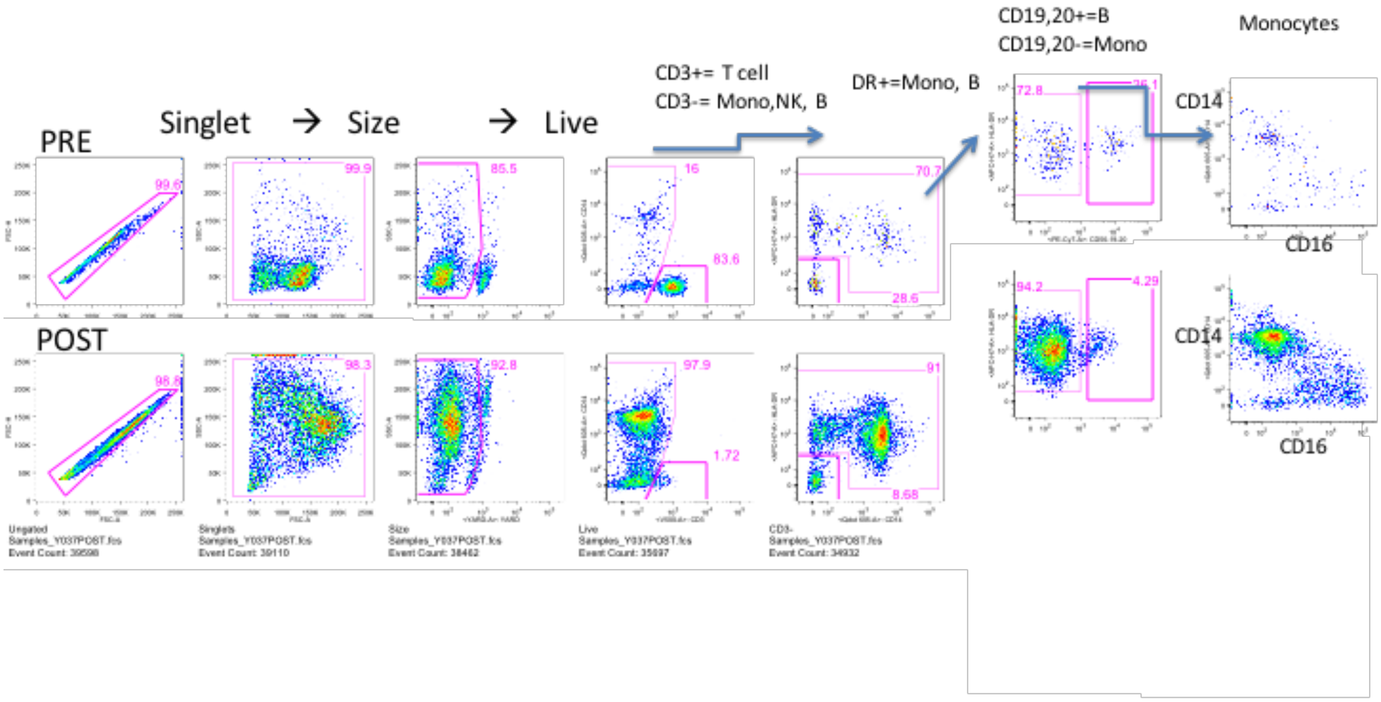

Supplementary Figure S1. Gating Strategy to confirm monocyte enrichment post-immunomagnetic enrichment. Aliquots of pre- (Top row) and post-immunomagnetic (Bottom row) negative selection enrichment PBMCs were analyzed for percent CD14(+)CD16(+) monocytes based on gating strategies previously described[1].

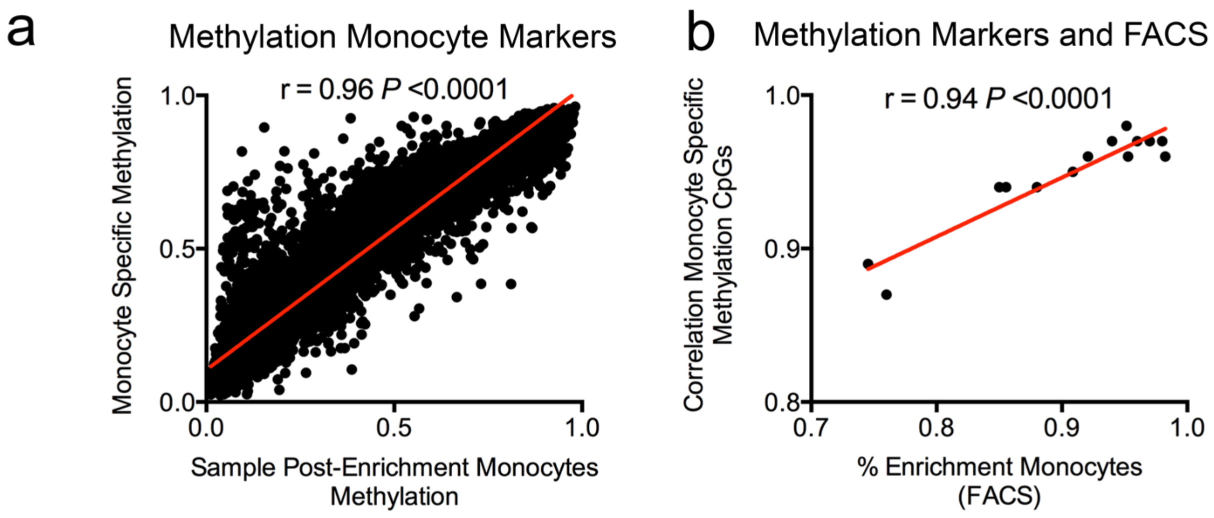

Supplementary Figure S2. Validation of monocyte cell enrichment by cell type-specific DNA methylation analysis. (a) Relationship between reference DNA methylation dataset for monocyte cell type and Post-enrichment DNA methylation for study sample. **(b)** Relationship between correlation based on monocyte cell-type specific analysis and % enrichment monocytes calculated by FACS analysis.

a

DML Monocyte		
Methylation Class	*Non-CI*	*CI*
RLL	0	5
RLR	0	2
RRL	4	0
RRR	3	0

b

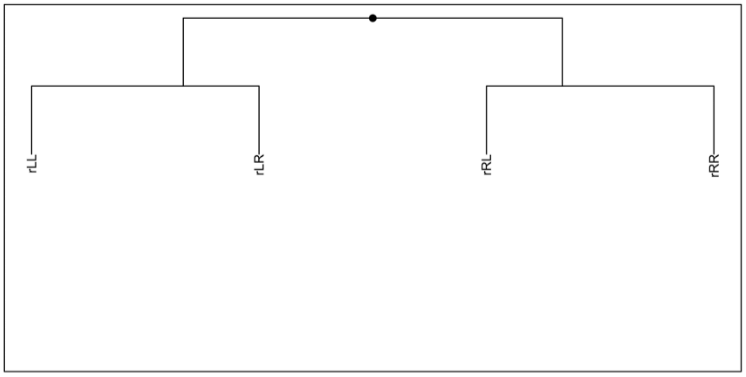

c

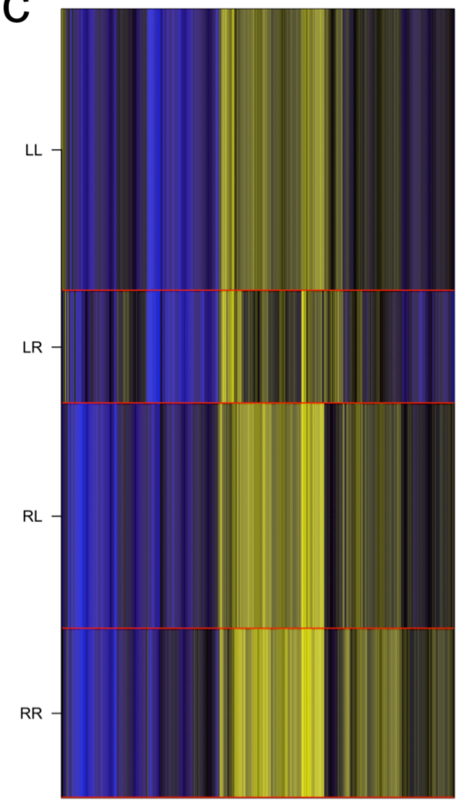

Supplementary Figure S3. Clustering of Differentially Methylated Loci in Monocytes with recursively partitioned mixture modeling (RPMM). (a) Methylation profile classes from RPMM of monocyte DML from Non-CI and CI participants. **(b)** RPMM tree dendogram showing 4 methylation classes clustering Non-CI and CI participants. **(c)** Columns represent differentially methylated CpG sites and rows represent the methylation classes. Row height displays number of observations residing in the class and red lines separates methylation classes. Blue indicates methylated and yellow indicates unmethylated.

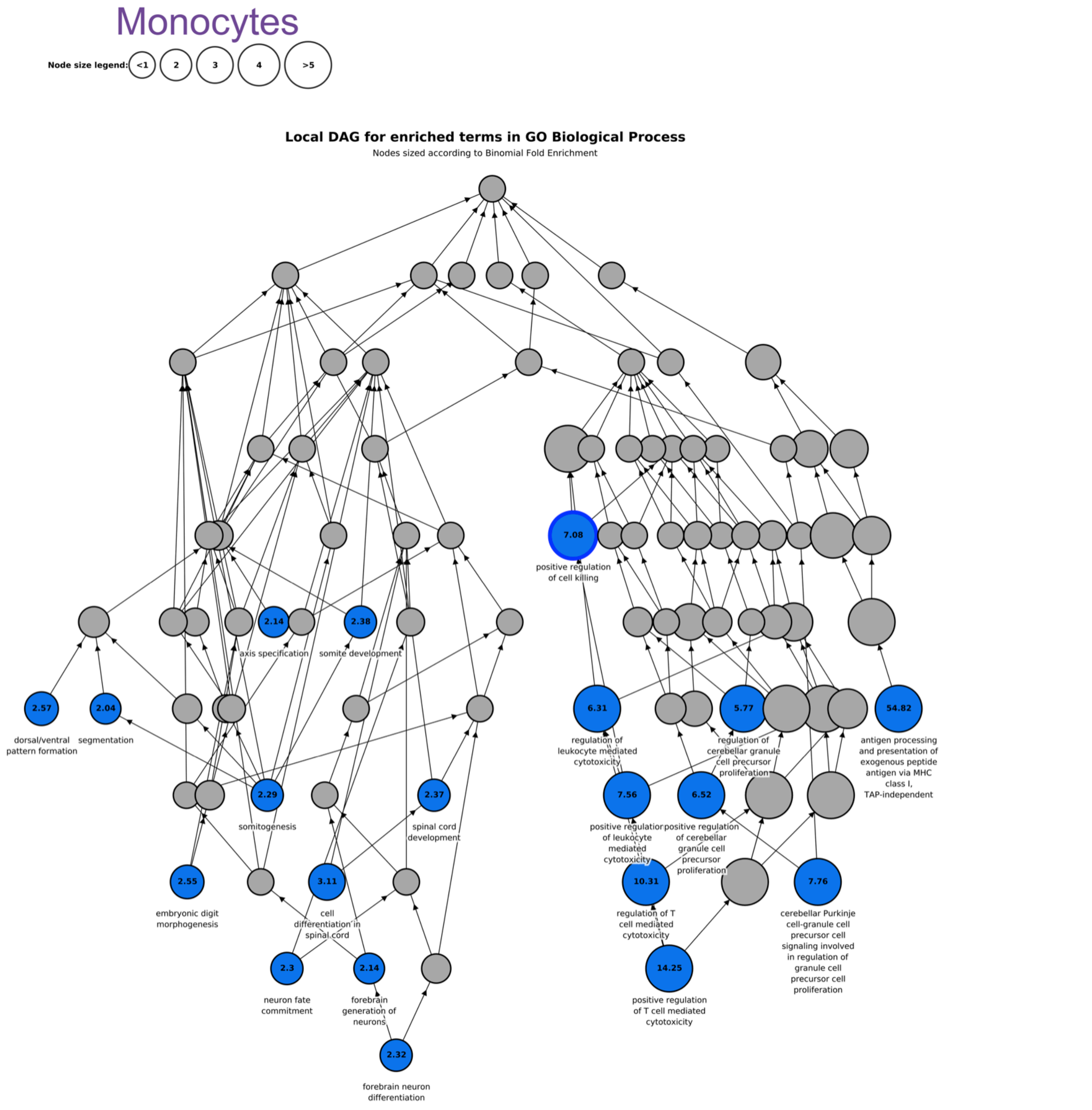

Supplementary Figure S4. GREAT analysis Gene Ontology Enrichment for Differentially Methylated Loci in Monocytes. HIV-associated cognitively impaired differentially methylated loci identified in monocytes were converted to genomic positions and analyzed using the GREAT tool[2]. Binomial fold enrichment presented in each node and enriched nodes term presented below.

a

DML Monocyte Profile				
Methylation Class	*Monocyte*		*T Cell*	
	Non-CI	*CI*	*Non-CI*	*CI*
RLL	2	2	0	0
RLR	0	5	0	0
RRL	5	0	0	0
RRR	0	0	3	4

b

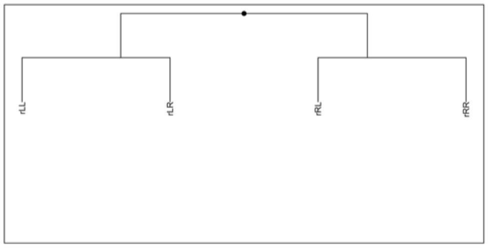

c

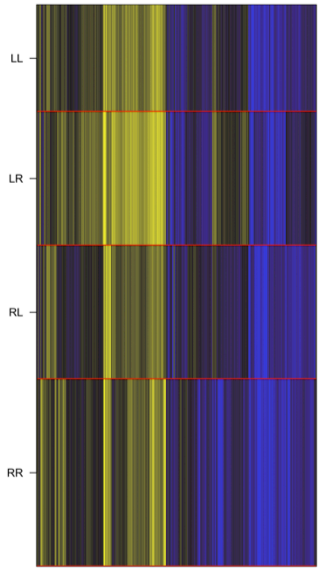

d

DML T Cell Profile				
Methylation Class	*Monocyte*		*T Cell*	
	Non-CI	*CI*	*Non-CI*	*CI*
RL	7	7	0	0
RRL	0	0	3	0
RRR	0	0	0	4

e

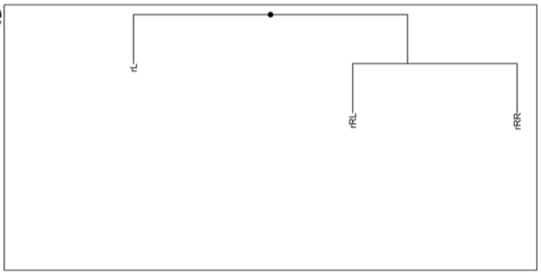

f

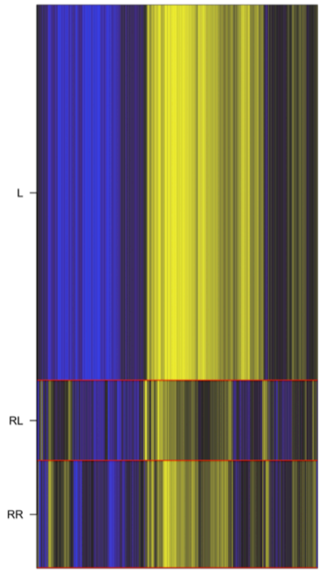

Supplementary Figure S5. CI-associated DML in monocytes stratifies CI in T cells with recursively partitioned mixture modeling (RPMM). (a) Methylation profile classes from RPMM analysis of monocyte DML profile from Non-CI and CI participants monocytes and T cells. **(b)** RPMM tree dendogram showing 4 methylation classes clustering Non-CI and CI participants. **(c)** Columns represent differentially methylated CpG sites and rows represent the methylation classes. Row height displays number of observations residing in the class and red lines separates methylation classes. Blue indicates methylated and yellow indicates unmethylated. **(d)** Methylation profile classes from RPMM analysis of CD8+ T Cell DML profile from Non-CI and CI participants monocytes and T cells. **(e)** RPMM tree dendogram showing 4 methylation classes clustering Non-CI and CI participants. **(f)** Columns represent differentially methylated CpG sites and rows represent the methylation classes. Row height displays number of observations residing in the class and red lines separates methylation classes. Blue indicates methylated and yellow indicates unmethylated.

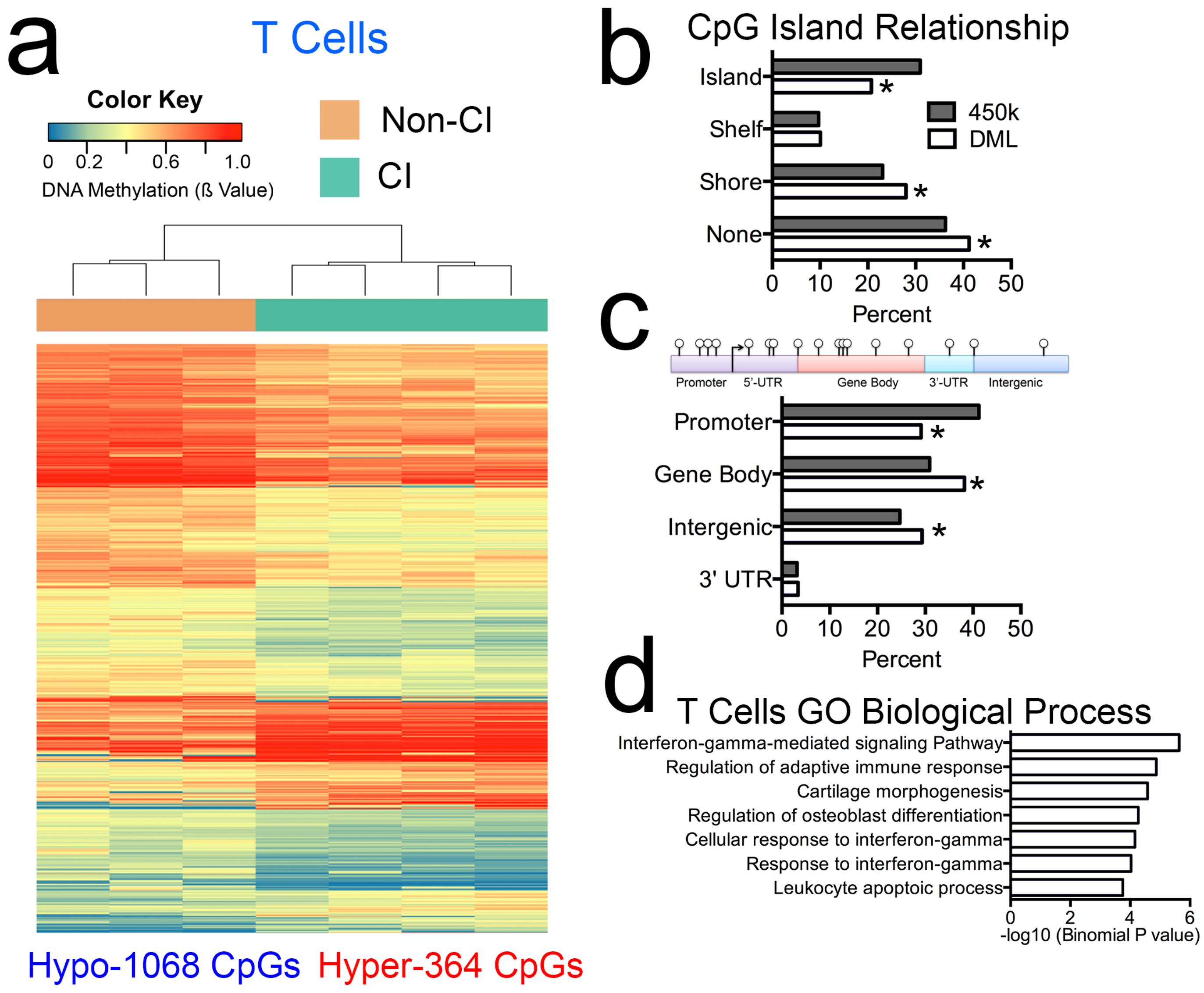

Supplementary Figure S6. HIV CI-associated DNA methylation differences in CD8+ T cells. (a) Heatmap displaying methylation levels of differentially methylated loci in Non-CI (orange) and CI (green) monocyte samples. The majority of these loci were hypo-methylated in CI compared to Non-CI. Unsupervised hierarchical clustering analysis (Manhattan distance, complete linkage method) above columns stratifies Non-CI and CI samples. Methylation values of each CpG displayed ranging from low methylation (0, blue) to high methylation (1, red). **(b)** Plot showing percent differentially methylated loci (white) located in CpG islands, shelves, shores, or none (Non-CpG island) compared to percent observed for all probes on the 450k array (grey). Significant enrichment for DML in CpG island shores compared to expected by chance. **(c)** Percent differentially methylated loci (white) located in gene promoters, gene bodies, intergenic regions, and 3’ UTR compared to percent observed for all probes on the 450k array (grey) shows significantly higher than expected gene body and intergenic location of loci compared to expected by chance. **(d)** GREAT analysis of DML displaying significant GO biological processes.

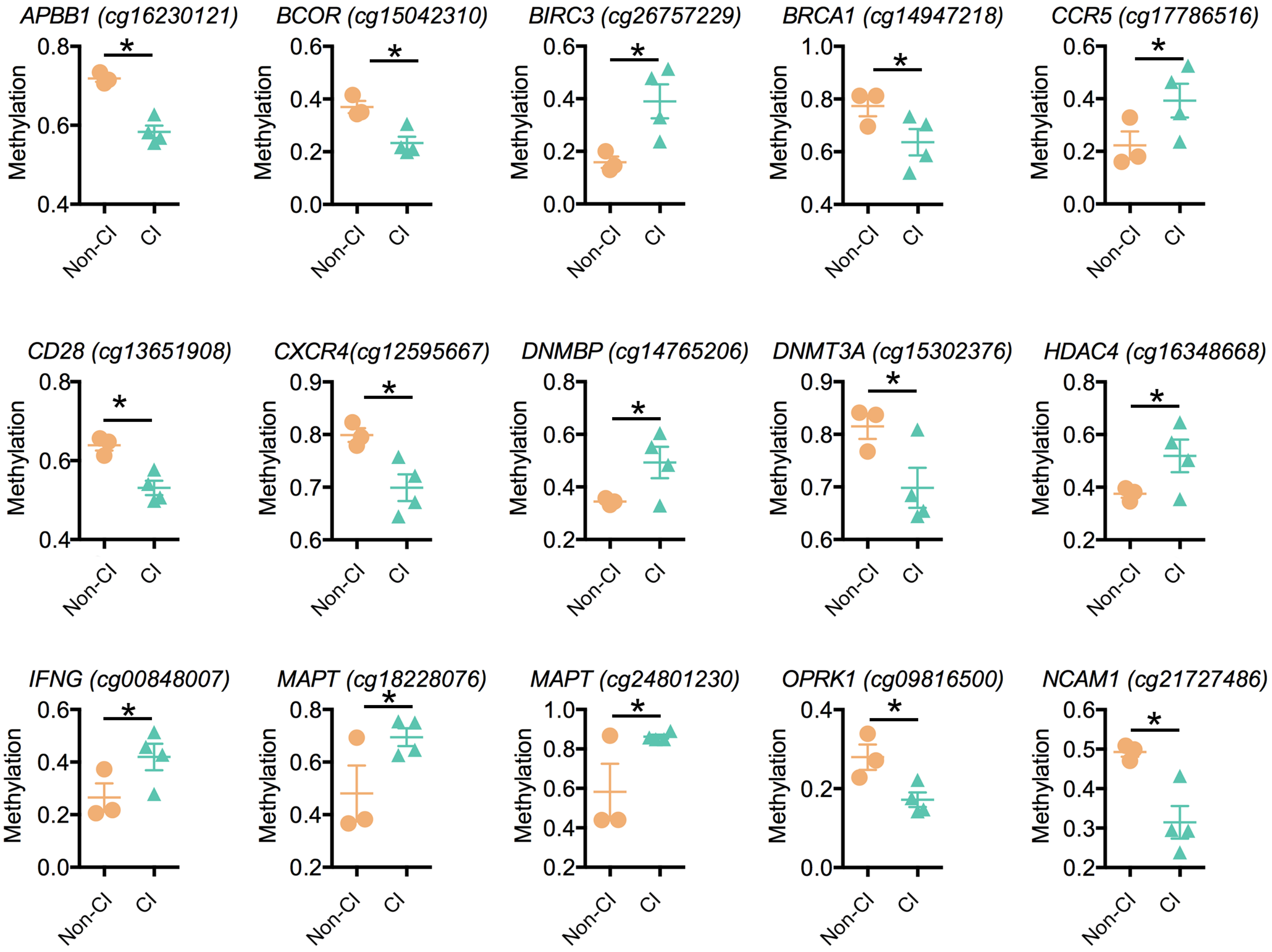

Supplementary Figure S7. HIV CI-associated DNA methylation differences in CD8+ T cells.
Differentially methylated loci in Non-CI and CI T cell samples located in genes related to central nervous system, interactions with HIV, and epigenetic mechanisms. * *P* <0.05

Supplemental Table S1. Cognitive Impaired (CI) and Non-CI Samples

Group	PID	Cognition
Non-CI	Y041S	Normal
	Y135S	Normal
	Y136C	Normal
	Y160L	Normal
	Y068	Normal
	Y078	Normal
	Y106	Normal
	Y028	Normal
	Y065	Normal
	Y103	Normal
CI	Y168Z	HAD
	Y211T	HAD
	Y213P	MC/MD
	Y037	MC/MD
	Y060	HAD
	Y113	MC/MD
	Y008	MC/MD
	Y082	MC/MD
	Y094	HAD
	Y105	HAD
	Y118	HAD

Supplemental Table S2. FACS cell frequency percent following CD14+CD16+ monocyte enrichment

Non-CI			Pre-Enrichment				Post-Enrichment					Monocyte Methylation Correlation
PID	Log Viral Load	Cell Count	Mono	T Cell	B Cell	NK Cell	Cell Count	Mono	T Cell	B Cell	NK Cell	
Y041S	1.68	13 M	24.4%	63.3%	3.1%	9.3%	2.25 M	93.9%	0.2%	0.4%	5.6%	0.97
Y136C	4.08	15 M	29.7%	64.9%	1.8%	3.7%	2.5 M	98.4%	0.4%	0.3%	1.0%	0.97
Y160L	1.68	4.7 M	10.6%	84.3%	2.0%	3.1%	550 K	97.4%	0.2%	0.7%	1.8%	0.97
Y068	3.56	4.4 M	12.4%	79.2%	4.0%	4.5%	800 K	85.1%	3.1%	6.6%	5.3%	0.94
Y106	4.09	4.9 M	41.7%	39.2%	11.9 %	7.2%	600 K	76.0%	7.7%	11.3%	5.0%	0.87
Y135S	2.25	9.6 M	20.2%	69.2%	2.3%	8.3%	800 K	95.9%	0.1%	0.5%	3.4%	0.97
Y078	1.69	6.6 M	6.0%	85.1%	4.6%	4.4%	525 K	88.1%	3.3%	5.1%	3.5%	0.94
Mean			20.7%	69.3%	4.2%	5.8%		90.7%	2.1%	3.6%	3.7%	
CI			Pre-Enrichment				Post-Enrichment					
Y168Z	1.68	22 M	15.0%	76.6%	3.0%	5.4%	2.45 M	92.1%	1.6%	1.3%	5.1%	0.96
Y211T	1.68	5.7 M	15.6%	73.9%	3.3%	7.2%	950 K	95.3%	1.2%	0.6%	2.9%	0.96
Y213	1.68	6.1 M	24.4%	67.7%	0.9%	7.0%	1.2 M	95.2%	0.1%	0.5%	4.3%	0.98
Y060	2.96	6.3 M	10.7%	80.9%	0.9%	2.8%	825 K	90.9%	2.4%	4.9%	1.8%	0.95
Y113	1.69	6.9 M	12.8%	77.7%	5.8%	3.8%	625 K	85.6%	0.8%	4.0%	9.6%	0.94
Y037	1.69	14 M	14.3%	78.0%	3.2%	4.5%	1.8 M	98.2%	0.1%	0.2%	1.5%	0.96
Y008	1.69	2.2 M	17.9%	60.8%	2.0%	7.9%	450 K	74.5%	9.4%	11.5%	4.6%	0.89
Mean			15.8%	73.7%	2.7%	5.5%		90.3%	2.2%	3.3%	4.3%	

Supplemental Table S3. Enrichr Gene List Analysis: GO Biological Process

Name	P-value	Combined Score
regulation of ion transmembrane transport (GO:0034765)	2.51E-07	18.0
regulation of transmembrane transport (GO:0034762)	5.19E-07	18.0
homophilic cell adhesion via plasma membrane adhesion molecules (GO:0007156)	3.24E-07	16.9
cell-cell adhesion (GO:0098609)	8.95E-07	16.7
cell-cell adhesion via plasma-membrane adhesion molecules (GO:0098742)	8.32E-07	16.7
single-organism behavior (GO:0044708)	8.97E-06	13.0
behavior (GO:0007610)	1.25E-05	12.6
neuron recognition (GO:0008038)	4.39E-05	10.8
locomotory behavior (GO:0007626)	2.89E-05	10.6
regulation of appetite (GO:0032098)	1.93E-04	8.2
adult behavior (GO:0030534)	9.83E-05	8.1
regulation of endothelial cell proliferation (GO:0001936)	1.32E-04	7.6
regulation of calcium ion transport (GO:0051924)	1.72E-04	7.6
positive regulation of cell projection organization (GO:0031346)	1.93E-04	7.3
regionalization (GO:0003002)	2.56E-04	7.2
cognition (GO:0050890)	3.01E-04	6.8
neuron migration (GO:0001764)	2.67E-04	6.6
pattern specification process (GO:0007389)	4.06E-04	6.4
positive regulation of calcium-mediated signaling (GO:0050850)	7.10E-04	5.8
regulation of metal ion transport (GO:0010959)	8.10E-04	5.6

Supplemental Table S4. Enrichr Gene List Analysis: GO Cellular Component

Name	P-value	Combined Score
synapse part (GO:0044456)	1.95E-05	12.0
ion channel complex (GO:0034702)	2.73E-04	8.0
extracellular matrix (GO:0031012)	1.70E-04	8.0
synapse (GO:0045202)	4.14E-04	7.9
synaptic vesicle (GO:0008021)	3.85E-04	7.8
transmembrane transporter complex (GO:1902495)	8.24E-04	7.1
transporter complex (GO:1990351)	9.87E-04	7.0
integral component of plasma membrane (GO:0005887)	7.32E-04	6.8
anchored component of membrane (GO:0031225)	1.46E-03	5.8
external side of plasma membrane (GO:0009897)	3.07E-03	5.2
proteinaceous extracellular matrix (GO:0005578)	2.64E-03	5.0
terminal bouton (GO:0043195)	3.53E-03	4.8
side of membrane (GO:0098552)	5.81E-03	4.3
axon part (GO:0033267)	6.30E-03	4.0
photoreceptor inner segment (GO:0001917)	5.03E-03	4.0
synaptic membrane (GO:0097060)	1.12E-02	3.5
collagen trimer (GO:0005581)	8.21E-03	3.5
cell surface (GO:0009986)	1.13E-02	3.4
perikaryon (GO:0043204)	7.87E-03	3.4
cation channel complex (GO:0034703)	1.13E-02	3.2

Supplemental Table S5. Enrichr Gene List Analysis: GO Molecular Function

Name	P-value	Combined Score
sulfur compound binding (GO:1901681)	3.92E-05	8.8
carboxylic acid binding (GO:0031406)	2.95E-04	6.8
organic acid binding (GO:0043177)	3.10E-04	6.8
calcium ion binding (GO:0005509)	5.91E-04	5.7
transforming growth factor beta binding (GO:0050431)	1.29E-03	5.5
structural constituent of muscle (GO:0008307)	9.92E-04	5.2
substrate-specific channel activity (GO:0022838)	2.16E-03	4.9
cell adhesion molecule binding (GO:0050839)	1.72E-03	4.7
passive transmembrane transporter activity (GO:0022803)	3.80E-03	4.6
channel activity (GO:0015267)	3.80E-03	4.6
transmembrane receptor protein kinase activity (GO:0019199)	1.70E-03	4.5
amino acid binding (GO:0016597)	2.31E-03	4.4
gated channel activity (GO:0022836)	4.72E-03	4.3
voltage-gated ion channel activity (GO:0005244)	4.27E-03	4.3
voltage-gated channel activity (GO:0022832)	4.27E-03	4.3
glycosaminoglycan binding (GO:0005539)	4.44E-03	4.1
neurotransmitter receptor activity (GO:0030594)	3.83E-03	4.0
phosphoric ester hydrolase activity (GO:0042578)	6.09E-03	4.0
heparin binding (GO:0008201)	4.08E-03	4.0
ion channel activity (GO:0005216)	7.38E-03	3.9

Supplemental Table S6. Monocyte DML: Loci Related to Inflammatory Response

Monocytes DMS: Inflammatory Response

Probe ID	Genomic Position	Gene	Gene Region	P-value	Mean CI	Mean Non-CI	Beta Diff.
cg24491749	chr4:107236485-107236486	*AIMP1*	TSS1500	0.01	0.48	0.64	0.16
cg00887153	chr16:71559593-71559594	*CHST4*	TSS1500	0.04	0.59	0.48	0.11
cg01360628	chr3:89156118-89156119	*EPHA3*	TSS1500	0.05	0.61	0.49	0.12
cg05463589	chr16:88706426-88706427	*IL17C*	Body	0.01	0.65	0.46	0.19
cg21593409	chr16:88706389-88706390	*IL17C*	Body	0.01	0.62	0.48	0.14
cg11931596	chr8:79674645-79674646	*IL7*	Body	0.04	0.38	0.27	0.11
cg12454167	chr3:186435060-186435061	*KNG1*	TSS200	0.01	0.77	0.64	0.13
cg13150801	chr6:6606065-6606066	*LY86*	Body	0.02	0.59	0.46	0.13
cg06823034	chr14:24780734-24780735	*LTB4R*	5'UTR	0.01	0.19	0.09	0.11
cg00831909	chr1:13909623-13909624	*PDPN*	TSS1500	0.02	0.56	0.44	0.13
cg19702271	chr6:46703495-46703496	*PLA2G7*	TSS1500	0.00	0.31	0.20	0.11
cg25503381	chr15:39871923-39871924	*THBS1*	TSS1500	0.01	0.77	0.89	0.12
cg08433504	chr15:39872071-39872072	*THBS1*	TSS1500	0.02	0.75	0.87	0.12
cg10078511	chr15:39872032-39872033	*THBS1*	TSS1500	0.02	0.73	0.83	0.10
cg26123256	chr2:27530670-27530671	*UCN*	Body	0.02	0.45	0.35	0.10

Supplemental Table S7. Cytokine Signaling in Immune System Genes Differentially Expressed between CI and Non-CI Monocytes

Gene Symbol	Mean RPM Non-CI	Mean RPM CI	P-value
CISH	29.84	14.20	1.37E-03
DDX58	105.69	54.01	3.75E-02
EGR1	888.81	1371.58	3.29E-02
HLA-F	29.68	15.26	2.64E-02
IFIT1	42.70	12.14	1.39E-02
IFIT2	164.80	65.84	2.29E-02
IFIT3	112.37	37.18	9.49E-03
IL1B	36.37	144.39	1.66E-02
IL2RB	53.62	25.95	3.38E-02
IRS2	73.33	138.86	1.76E-02
JAK3	215.10	139.92	4.54E-02
NR4A2	13.30	52.68	1.18E-04
NUP50	72.35	46.56	2.32E-02
OAS2	171.45	81.92	2.25E-02
OAS3	453.95	215.73	4.67E-02
PELI1	82.05	169.82	5.45E-03
RIPK2	62.47	119.02	7.09E-03
RSAD2	39.80	11.54	1.33E-02
STAT2	597.09	343.93	1.03E-02
TRIM21	48.64	28.57	2.89E-02
TRIM26	53.82	36.53	4.79E-02
UBE2E1	45.47	23.77	1.17E-02

Supplemental Table S8. Cell Type Specific DNA Methylation T Cell Enrichment Analysis Results

Non-CI	T Cell Methylation Correlation
Y028	0.89
Y065	0.86
Y103	0.89
**CI**	
Y082	0.93
Y094	0.93
Y105	0.90
Y118	0.92

Supplementary References

1 Ndhlovu, L. C. *et al.* Loss of CCR2 expressing non-classical monocytes are associated with cognitive impairment in antiretroviral therapy-naive HIV-infected Thais. *Journal of neuroimmunology* **288**, 25-33, doi:10.1016/j.jneuroim.2015.08.020 (2015).

2 McLean, C. Y. *et al.* GREAT improves functional interpretation of cis-regulatory regions. *Nature biotechnology* **28**, 495-501, doi:10.1038/nbt.1630 (2010).
